# Supplementary material for: Split luciferase-based assay to detect botulinum neurotoxins using hiPSC-derived motor neurons
Source: Commun Biol. 2023 Jan 30;6:122. doi: 10.1038/s42003-023-04495-w (PMC9886929; doi:10.1038/s42003-023-04495-w)
Supplement: Supplementary file 2 — Supplementary information [file 42003_2023_4495_MOESM2_ESM.pdf]

Supplementary Figure 1

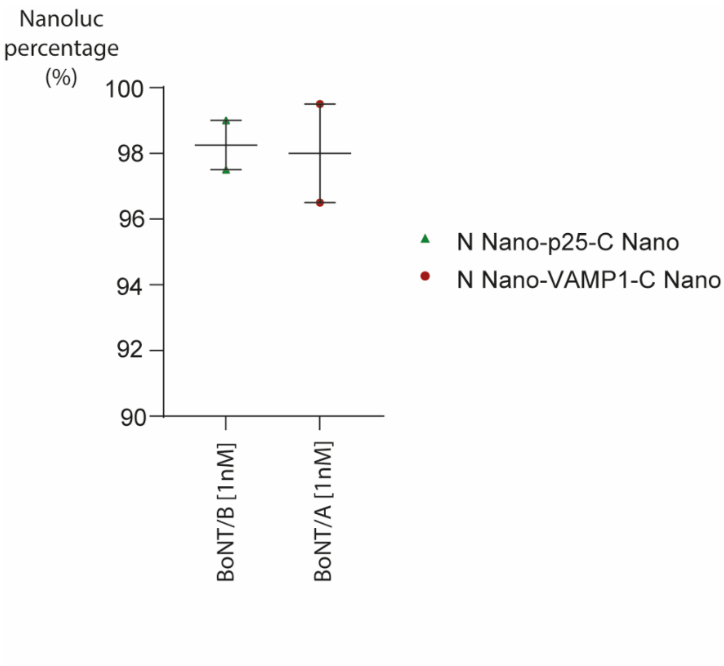

Negative control of acellular experiment

BoNT/A was mixed with Nnano-vp1-Cnano sensor protein while BoNT/B was mixed with Nnano-p25-Cnano sensor protein. Experiment was done twice in duplicate.

Supplementary Figure 2

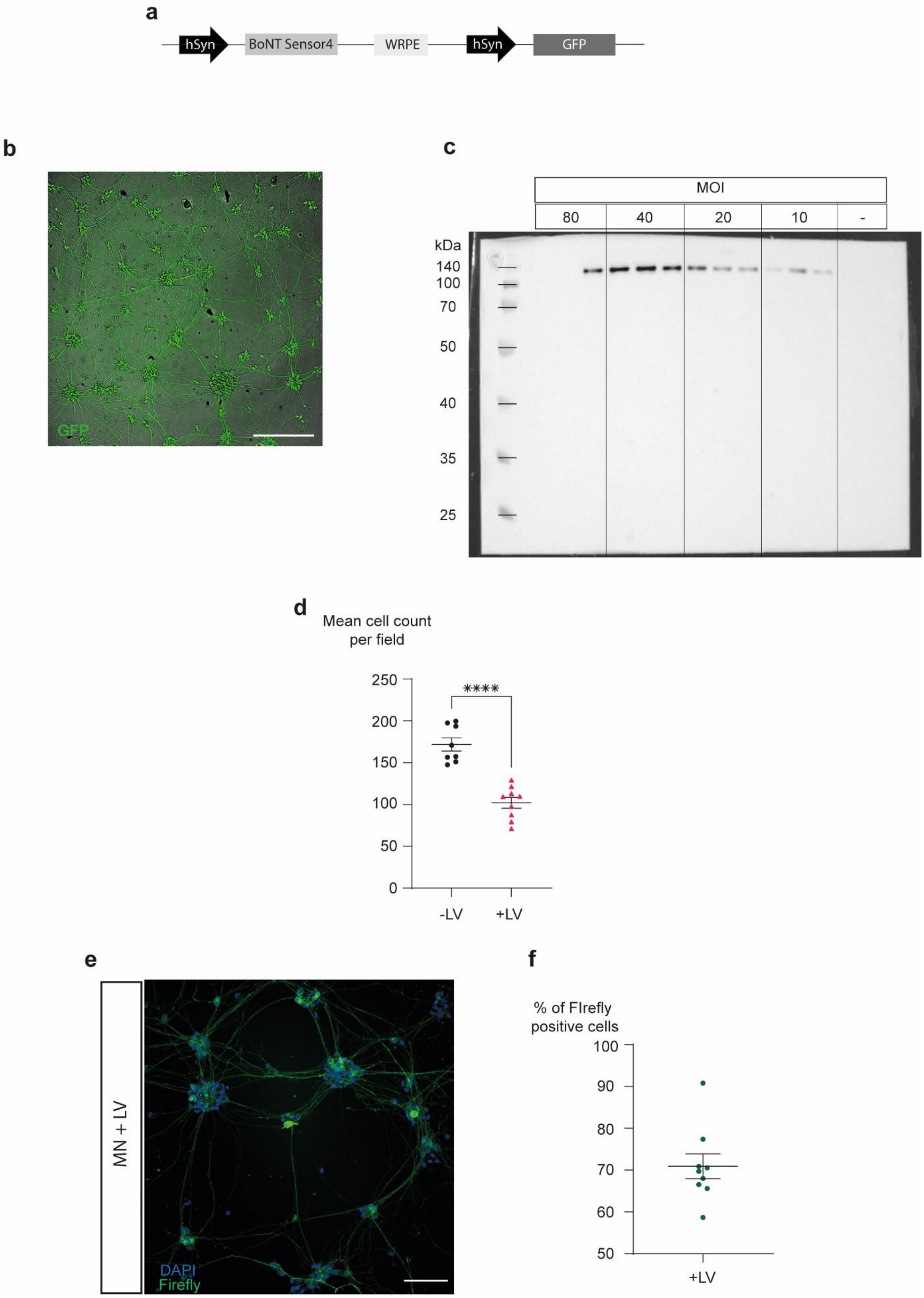

Impact of LV transduction on MN derived from hIPSC

a Schematic drawing of lentiviral vector containing two hSYN promoter/WRPE cassettes for constitutive expression of BoNT sensor4 and GFP in neurons. **b** iCellMNs transduced with dual-hSyn promotor lentivirus, 12 days after transduction (MOI 40). Representative image of transduced neurons expressing GFP (green). Scale bar 100 µm. **c** Full immunoblot of iCellMN lysate showing expression of BoNT sensor4 construct, 12 days after transduction with different MOI. The polypeptide is detected by an antibody against Firefly Luciferase. **d** Mean Dapi positive cells per field showing LV impact on cell viability (40% decrease). The percentage of DAPI^{+} cells in the indicated conditions represented as mean of 8 wells per conditions (≥1000 cells were counted per field **e-f** Lentiviral transduction (MOI 16) rate on MN 6 d after transfection. **e** Immunostaining of DAPI (blue) and Firefly (green) in iCellMN. Scale bar 100 µm. **f** Graph showing quantification of the percentage of Firefly^{+} MNs. This percentage is represented as mean of 8 wells per conditions (≥1000 cells were counted per well).
Statistical two-tailed Student's t-tests: *P<0.05; **P<0.01; ***P<0.001; NS, P>0.05 not significant (NS). Error bars show SEM.

Supplementary Figure 3

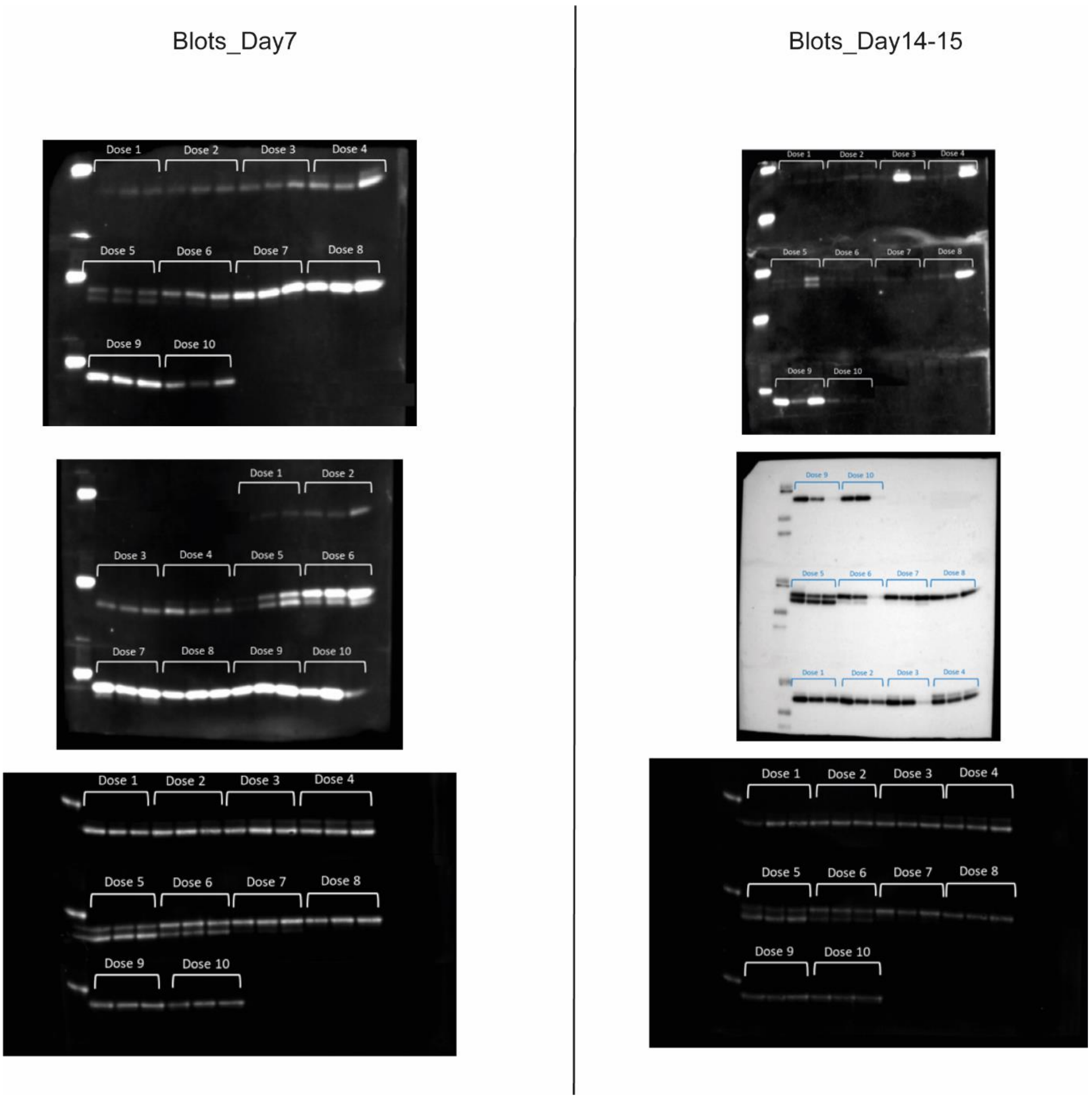

Full immunoblot showing the cleavage of SNAP25 after 24 hours treatment of BoNT/A at different doses. BoNT treatment was done after 7 days or two weeks of MN maturation. Dose 1 corresponds to no toxin added and Dose 10 is the highest dose (=1nM). We use an anti-SNAP25 antibody that recognize cleaved and uncleaved forms of SNAP-25.

Supplementary Figure 4

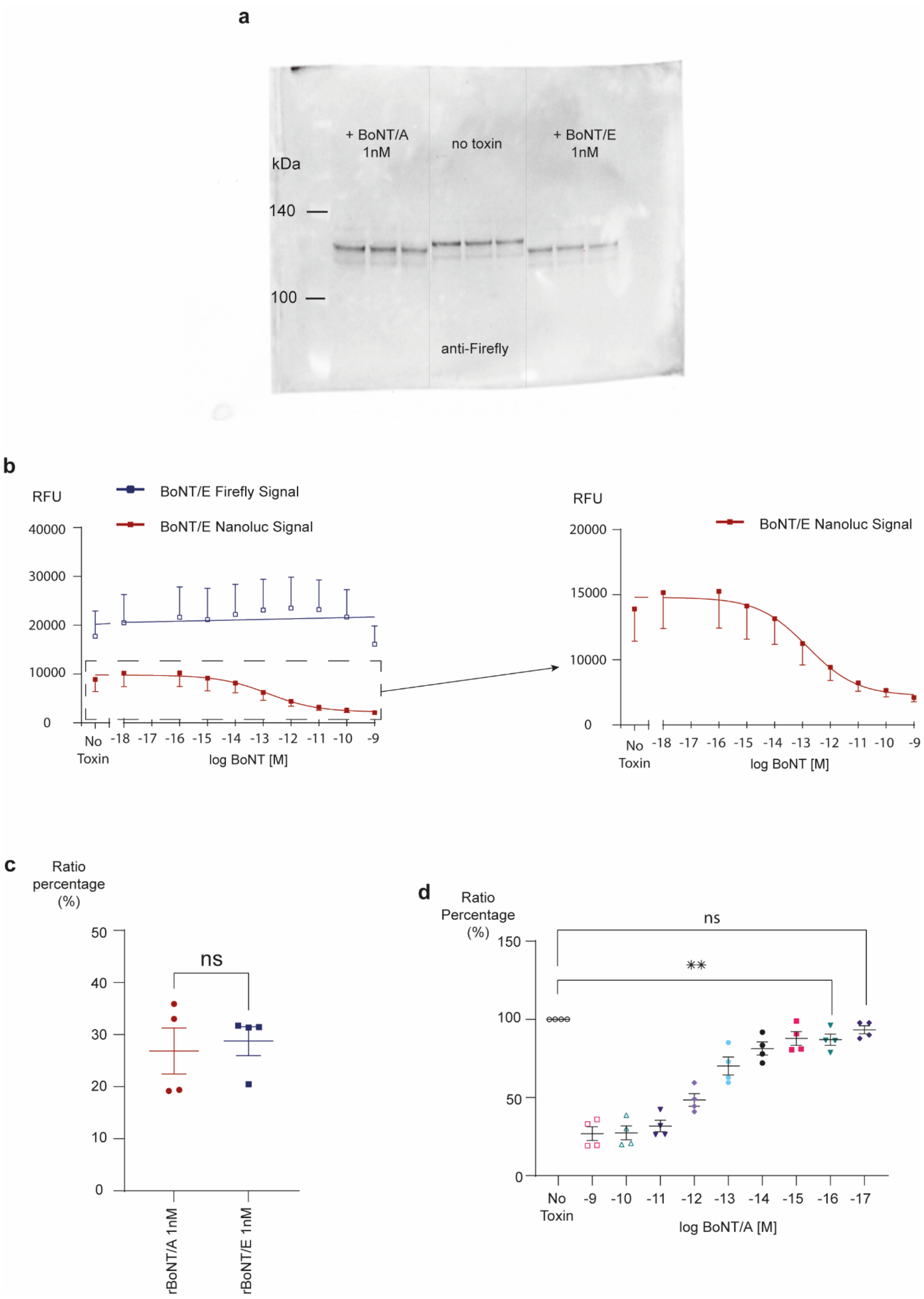

Cell-based assay using iCell MNs with BoNT sensor4.

a Full blot of iCell MN lysate showing cleavage of the Nanoluc sensor, 9 days after transduction, with the maximal concentration of BoNT/A and E. An antibody against Firefly luciferase detects the polypeptide. **b** Graph showing luminescent variation during BoNT treatment. As illustration, we show here a BoNT/E treatment. The Firefly signal (blue circles) stay stable although the Nanoluc signal (red squares) decrease when toxin concentration increase. **c** Graph of the ratio percentage obtained with the highest dose of toxin (=Rtox max) for BoNT/A and E. Data points are mean of n=4 independent experiments in triplicate. **d** Same graph for BoNT/A treatment as Figure 3B but replotted to highlight the limit of detection of the assay. Ratio percentage are plotted as individual values for each independent experiments and mean (n=4 experiments performed in triplicates).
Statistical ordinary one-way ANOVA multiple comparisons: *$P<0.05$; **$P<0.01$; ***$P<0.001$; ****$P<0.0001$; NS, $P>0.05$ not significant (NS). Error bars show SEM.

Supplementary Figure 5

Calculation exemple

Firefly signal = FS

LV	LV-BoNT sensor 4-GFP										
Tox_treatment rBoNT/E	1nM	100pM	10pM	1pM	0,1pM	0,01pM	1fM	0,1fM	0,01fM	-	Mean $FS_{w/o\ tox}$
FS [RLU]	44035	53835	57598	58272	60294	55659	59747	56564	52543	48514	44266
	33201	49065	54336	54289	53497	52872	52444	51191	49089	44582	
	24822	46916	51428	55963	52554	53313	49447	52895	48616	39701	

NanoLuc signal = NS

LV	LV-BoNT sensor 4-GFP										
Tox_treatment rBoNT/E	1nM	100pM	10pM	1pM	0,1pM	0,01pM	1fM	0,1fM	0,01fM	-	Mean $NS_{w/o\ tox}$
NS [RLU]	5002	5061	6878	10640	15894	20312	26023	26895	26619	24899	21295
	2593	5033	6293	8529	13545	17786	21728	23452	24460	22204	
	2531	5144	6056	9395	14581	16475	18151	23515	21657	16781	

Luminescence ratio =LR

LV	LV-BoNT sensor 4-GFP											
Tox_treatment rBoNT/E	1nM	100pM	10pM	1pM	0,1pM	0,01pM	1fM	0,1fM	0,01fM	-	Mean $LR_{w/o\ tox}$	Mean $LR_{w\ tox\ max}$
LR = NS/FS	0,1136	0,0940	0,1194	0,1826	0,2636	0,3649	0,4356	0,4755	0,5066	0,5132	0,4780	0,0979
	0,0781	0,1026	0,1158	0,1571	0,2532	0,3364	0,4143	0,4581	0,4983	0,4980		
	0,1020	0,1096	0,1178	0,1679	0,2774	0,3090	0,3671	0,4446	0,4455	0,4227		

Ratio percentage = RP

LV	LV-BoNT sensor 4-GFP											
Tox_treatment rBoNT/E	1nM	100pM	10pM	1pM	0,1pM	0,01pM	1fM	0,1fM	0,01fM	-	Mean $RP_{w/o\ tox}$	Mean $RP_{w\ tox\ max}$
$((NS/FS)/(MLR_{w/o\ tox}))*100$	23,7645	19,6677	24,9826	38,2001	55,1495	76,3483	91,1221	99,4749	105,9886	107,3735	100,0000	20,4787
	16,3393	21,4604	24,2299	32,8676	52,9702	70,3777	86,6775	95,8448	104,2448	104,1967		
	21,3323	22,9384	24,6359	35,1219	58,0449	64,6509	76,7968	93,0064	93,1969	88,4298		

Table illustrating the way of the ratio percentage was calculated in our assay.

Supplementary Figure 6

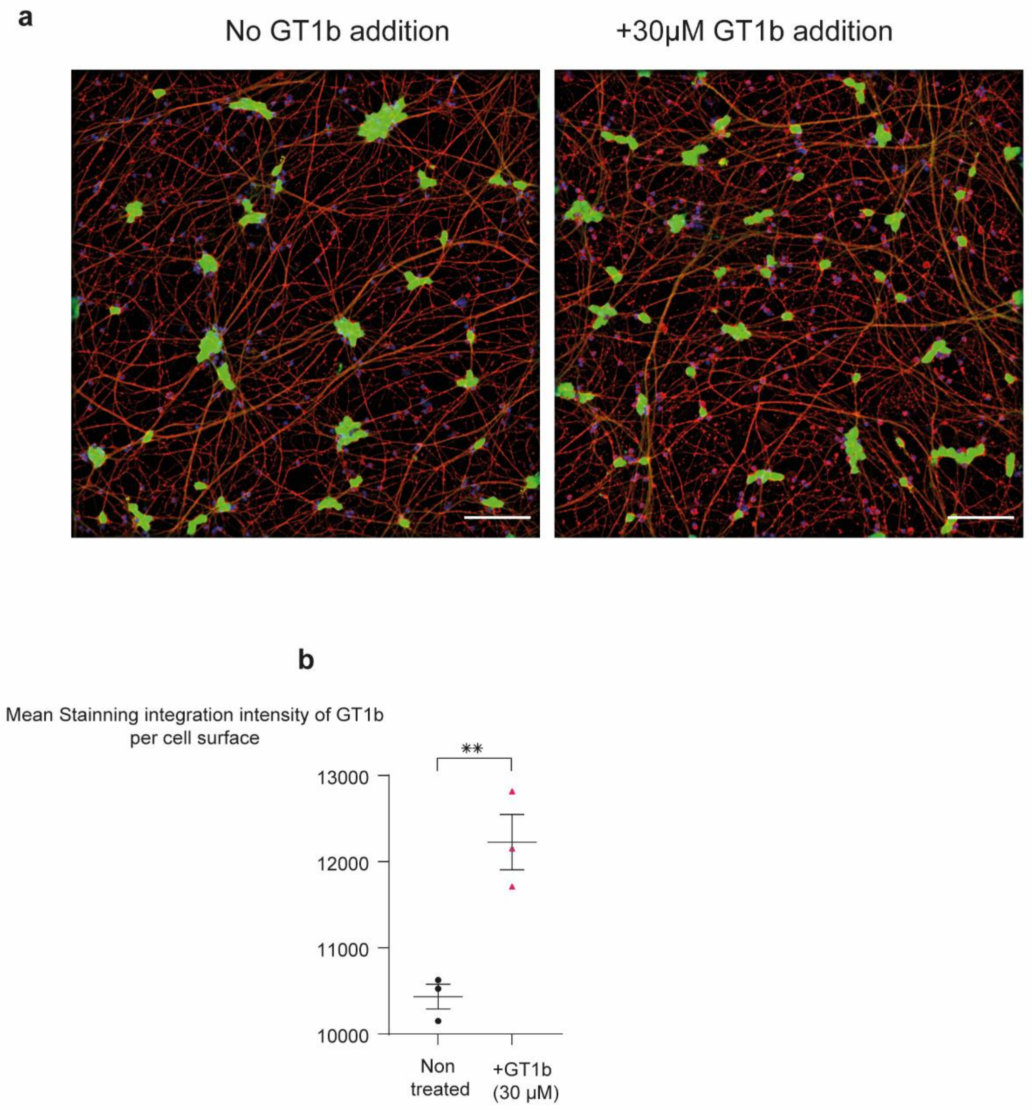

Quantification of GT1b ganglioside

Six days after thawing, GT1b (30µM) was added to the iCell MNs. 48h later, this media was removed, the cells were fixed and an immunostaining was performed. **a** The cell surface was stained by HCS CellMask™ Green Stain (H32714, Thermo Fisher Scientific, 1/5000) (green on picture) and an antibody against Gt1b

(MAB5608, Merck Millipore, 1/500) was also used (red). Scale bar 100 µm. **b** Graph of the quantification of ganglioside intensity normalized to the total cell area. For cell surface gangliosides quantification, the DAPI, CellMask™ (which stain the plasma membrane) and GT1b signal have been quantified using the multiple wavelength cell scoring module of the ImageXpress Micro Confocal High-Content Imaging System (Molecular Devices, San José, CA, USA). More precisely, we first determined the colocalization between these stainings. Then, after signal quantification of each channel, the integrated intensity signal of GT1b have been normalized to the total cell area using CellMask™ positive area. Gangliosides quantifications are plotted as the mean integrated intensity staining of GT1b normalized to the total cell surface and mean of three different well per condition.
Statistical ordinary one-way ANOVA multiple comparisons: *P<0.05; **P<0.01; ***P<0.001; ****P<0.0001; NS, P>0.05 not significant (NS). Error bars show SEM.
