# Supplementary material for: Split luciferase-based assay to detect botulinum neurotoxins using hiPSC-derived motor neurons
Source: Commun Biol. 2023 Jan 30;6:122. doi: 10.1038/s42003-023-04495-w (PMC9886929; doi:10.1038/s42003-023-04495-w)
Supplement: Supplementary file 3 — Description of Additional Supplementary Files [file 42003_2023_4495_MOESM3_ESM.pdf]

Description of Additional Supplementary Files

File name: Supplementary Data 1-16

Description:

Supplementary Data 1: Source file for Figure 1c.

Supplementary Data 2: Source file for Figure 1d.

Supplementary Data 3: Source file for Figure 2b.

Supplementary Data 4: Source file for Figure 2c.

Supplementary Data 5: Source file for Figure 2e.

Supplementary Data 6: Source file for Figure 3b.

Supplementary Data 7: Source file for Figure 3c.

Supplementary Data 8: Source file for Figure 4a.

Supplementary Data 9: Source file for Figure 4b.

Supplementary Data 10: Source file for Supplementary Figure 1.

Supplementary Data 11: Source file for Supplementary Figure 2d.

Supplementary Data 12: Source file for Supplementary Figure 2f.

Supplementary Data 13: Source file for Supplementary Figure 4b.

Supplementary Data 14: Source file for Supplementary Figure 4c.

Supplementary Data 15: Source file for Supplementary Figure 4d.

Supplementary Data 16: Source file for Supplementary Figure 6b.
