# Supplementary material for: Split luciferase-based assay to detect botulinum neurotoxins using hiPSC-derived motor neurons
Source: Commun Biol. 2023 Jan 30;6:122. doi: 10.1038/s42003-023-04495-w (PMC9886929; doi:10.1038/s42003-023-04495-w)
Supplement: Supplementary file 5 — Reporting Summary [file 42003_2023_4495_MOESM5_ESM.pdf]

nature portfolio

Corresponding author(s): camille.nicoleau@ipsen.com

Last updated by author(s): 8/17/2021

Reporting Summary

Nature Portfolio wishes to improve the reproducibility of the work that we publish. This form provides structure for consistency and transparency in reporting. For further information on Nature Portfolio policies, see our Editorial Policies and the Editorial Policy Checklist.

Statistics

For all statistical analyses, confirm that the following items are present in the figure legend, table legend, main text, or Methods section.

n/a	Confirmed	
☐	☒	The exact sample size (n) for each experimental group/condition, given as a discrete number and unit of measurement
☐	☒	A statement on whether measurements were taken from distinct samples or whether the same sample was measured repeatedly
☐	☒	The statistical test(s) used AND whether they are one- or two-sided<br>*Only common tests should be described solely by name; describe more complex techniques in the Methods section.*
☒	☐	A description of all covariates tested
☒	☐	A description of any assumptions or corrections, such as tests of normality and adjustment for multiple comparisons
☐	☒	A full description of the statistical parameters including central tendency (e.g. means) or other basic estimates (e.g. regression coefficient) AND variation (e.g. standard deviation) or associated estimates of uncertainty (e.g. confidence intervals)
☐	☒	For null hypothesis testing, the test statistic (e.g. F, t, r) with confidence intervals, effect sizes, degrees of freedom and P value noted<br>*Give P values as exact values whenever suitable.*
☒	☐	For Bayesian analysis, information on the choice of priors and Markov chain Monte Carlo settings
☒	☐	For hierarchical and complex designs, identification of the appropriate level for tests and full reporting of outcomes
☐	☐	Estimates of effect sizes (e.g. Cohen's d, Pearson's r), indicating how they were calculated

Our web collection on statistics for biologists contains articles on many of the points above.

Software and code

Policy information about availability of computer code

Data collection	not applicable
Data analysis	not applicable

For manuscripts utilizing custom algorithms or software that are central to the research but not yet described in published literature, software must be made available to editors and reviewers. We strongly encourage code deposition in a community repository (e.g. GitHub). See the Nature Portfolio guidelines for submitting code & software for further information.

Data

Policy information about availability of data

All manuscripts must include a data availability statement. This statement should provide the following information, where applicable:

- Accession codes, unique identifiers, or web links for publicly available datasets
- A description of any restrictions on data availability
- For clinical datasets or third party data, please ensure that the statement adheres to our policy

not applicable

Field-specific reporting

Please select the one below that is the best fit for your research. If you are not sure, read the appropriate sections before making your selection.

☒ Life sciences ☐ Behavioural & social sciences ☐ Ecological, evolutionary & environmental sciences

For a reference copy of the document with all sections, see nature.com/documents/nr-reporting-summary-flat.pdf

Life sciences study design

All studies must disclose on these points even when the disclosure is negative.

Sample size	No sample-size calculation was performed. For eaxh experiment cells were plated on a 96 well-plate. Each conditions tested were done in three wells (triplicate) then the experiment were repeated at least three time
Data exclusions	We exclude data when we noticed that there were some cell detachment occuring during cell treatment .
Replication	All experiment were independantly replicated at least 3 time
Randomization	The experiments were not randomized. To avoid contamination and facilitate toxin treatment, drug was applied always on the same way.
Blinding	Blinding was not done because the result was read by an unbiased machine

Reporting for specific materials, systems and methods

We require information from authors about some types of materials, experimental systems and methods used in many studies. Here, indicate whether each material, system or method listed is relevant to your study. If you are not sure if a list item applies to your research, read the appropriate section before selecting a response.

Materials & experimental systems

n/a	Involved in the study	
☐	☒	Antibodies
☐	☒	Eukaryotic cell lines
☒	☐	Palaeontology and archaeology
☐	☒	Animals and other organisms
☒	☐	Human research participants
☒	☐	Clinical data
☒	☐	Dual use research of concern

Methods

n/a	Involved in the study	
☒	☐	ChIP-seq
☒	☐	Flow cytometry
☒	☐	MRI-based neuroimaging

Antibodies

Antibodies used	For Western blot cleavage assay (Figure 2C): anti-SNAP25 antibody (S9684, Sigma-Aldrich, 1/2000) and horseradish peroxidase–conjugated secondary anti-rabbit (A6154, Sigma-Aldrich, 1/2000) antibody.<br>For immunostaining (Figure 2D): Goat Anti-Islet1 (GT15051, Neuromics Inc., Edina, MN, USA; 1:200) and Alexa 647 Donkey anti-Goat (A21447, Thermo Fisher Scientific).
Validation	These antibodies have been validated by colleagues and others.<br>Ref: Duchesne de Lamotte J, Roqueviere S, Gautier H, Raban E, Bouré C, Fonfria E, Krupp J & Nicoleau C (2021) hiPSC-Derived Neurons Provide a Robust and Physiologically Relevant In Vitro Platform to Test Botulinum Neurotoxins. Front Pharmacol 11, 617867

Eukaryotic cell lines

Policy information about cell lines

Cell line source(s)	Frozen iCell Motor Neurons provided by FujiFilm Cellular Dynamics International (FCDI, Madison, WI, USA) and Primary cell culture from mice
Authentication	Full characterization of FCDI Motor Neurons published by us (Duchesne de Lamotte J, et al., 2021 Front Pharmacol 11, 617867), also available through FCDI website
Mycoplasma contamination	cell lines were not tested for mycoplasma

Commonly misidentified lines (See ICLAC register)	No misidentified cell lines were used in this study

Animals and other organisms

Policy information about studies involving animals; ARRIVE guidelines recommended for reporting animal research

Laboratory animals	Sprague Dawley rats purchased from Charles River
Wild animals	not applicable
Field-collected samples	not applicable
Ethics oversight	This study was carried out in strict accordance with the recommendations in the Guide for the Care and Use of Laboratory Animals of the National Institutes of Health. The protocol was approved by the Standing Committee on Animals of Harvard Medical School (Permit Number: 04619). All efforts were made to minimize suffering of animals

Note that full information on the approval of the study protocol must also be provided in the manuscript.
